# An Investigation into the Use and Meaning of Parkinson's Disease Clinical Scale Scores

**DOI:** 10.1155/2021/1765220

**Published:** 2021-05-29

**Authors:** Renee M. Hendricks, Mohammad T. Khasawneh

**Affiliations:** Department of Systems Science and Industrial Engineering, Binghamton University, P.O. Box 6000, Binghamton, NY 13902-6000, USA

## Abstract

Parkinson's disease (PD) is the second most common, neurodegenerative disorder. It is a chronic, disabling, and progressive disease, and no treatment stops its progression. Rating scales are utilized to quantify PD progression and severity. The most conventional scale is the Unified Parkinson's Disease Rating Scale (UPDRS) and its modified version, Movement Disorder Society- (MDS-) UPDRS. An analytical investigation into the use and meaning of these clinical scale scores was conducted to determine if gaps exist in quantifying disease progression and severity. A series of discrepancies were identified including confusion among patients regarding the score meaning and misuse of the scores among clinicians and researchers to define disease progression. The scales are of an ordinal type and hence the resulting scores are ordinal, not providing a quantifiable progression nor severity level, but a categorical value and survey total. The knowledge that the scores are ordinal and the scales are subjective is mentioned in very limited publications, not the focus of these papers, but a brief introduction and a thoroughly researched, analytical investigation into the scales and scores have not been found. Therefore, the continuous misunderstanding and misuse of these scales and resulting scores warrant a comprehensive assessment and evaluation of these scales and scores to identify the gaps.

## 1. Introduction

Parkinson's disease (PD) is a chronic and progressive disease and no treatment stops its progression. It is widely known that PD is prevalent in the aging population with some clinicians reporting a mean age of onset in the early 60s [1]. Standardized scales attempt to quantify disease progression and severity, symptoms, and quality of life [2]. The most common and globally recognized scale to assess disease severity is the Unified Parkinson Disease Rating Scale (UPDRS) [3]. In 2008, the Movement Disorder Society (MDS) developed and published the revised UPDRS scale, referred to as MDS-UPDRS [4].

An analytical investigation into the use and meaning of these clinical scales' scores was conducted to determine if any gaps exist in quantifying disease progression and severity. Both qualitative and quantitative analyses were applied. The objectives of this study were to determine if the scales and scores are linear or nonlinear, and if the score values are of numerical or categorical type. These determinations will provide insight into the score meanings and the appropriate analyses that can be applied. In addition, studies that utilized the clinical scores for describing patient groups or subtypes will be reviewed and critiqued. Furthermore, any information discovered regarding patient or practitioner judgements of the scales or scores will also be reported. The implications of incorrectly defining and calculating Parkinson's disease progression through these scales and scores may cause confusion regarding the score(s) meanings and misuse in evaluating and defining disease progression. The intent of this paper is that the results of analytical analysis will change the direction in correctly defining and evaluating PD symptoms, progression, and severity.

## 2. Materials and Methods

The UPDRS and MDS-UPDRS scales were obtained, reviewed, and critiqued for this study. Qualitative and quantitate analyses were conducted on the UPDRS and MDS-UPDRS. For the qualitative analysis, content analysis was conducted to analyze the meanings, relationships, and interpretations of the scales and scores. For the quantitate analysis, a section of the scale scores was computed and reviewed. In addition, a review of studies which described patient subtypes or clusters with these scores was critiqued. In addition, a literature review was conducted to determine if published sources contained similar findings.

## 3. Qualitative Results and Discussion

This section provides a commentary of the UPDRS and MDS-UPDRS questions and score discoveries.

### 3.1. UPDRS

**Figure 1:**
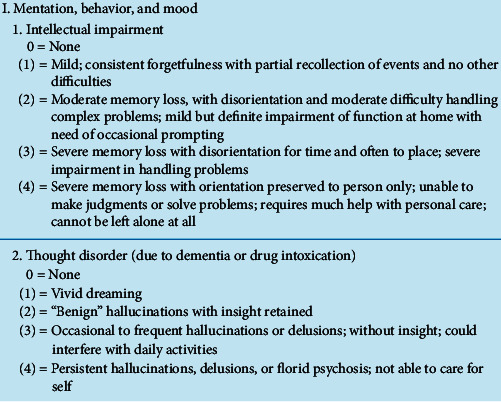
UPDRS Part I questions 1 and 2 [2].

The UPDRS was developed in 1987 to incorporate elements from existing scales and provide a comprehensive tool to capture and assess multiple aspects of PD, including motor disability, motor impairment, mental dysfunction and mood, and treatment-related motor and nonmotor complications [5]. The UPDRS contains four parts. Part I focuses on nonmotor symptoms, such as dementia, depression, and psychosis. Part II focuses on the patient's ability to perform daily activities including dressing, grooming, and using utensils. Part III is rated by a clinician and measures the motor features of speech, facial expression, tremor, tone, movement slowness in the hands and legs, walking, and balance. Part IV measures the complications of treatment [2].  Figure 1 displays questions 1 and 2 from Part I. It is a 5-point scale, with choices ranging from 0 to 4.

Utilizing the two UPDRS questions in Figure 1, one can initially observe the following:

- Response choices are in a 5-point range (0–4), similar to a Likert scale
- Choice meanings are different (1 = mild or vivid dreaming based on question)
- Choices can list a variety of symptoms and descriptions
- Choice numbers do not correspond to a symptom stage

Commonly utilized scales include nominal, ordinal, and interval. An example nominal scale would consist of arbitrarily assigning the number 1 to male and number 2 to female. The numbers do not have any meaning, but are simply utilized to categorize subjects into groups for counting. An ordinal scale can be utilized when asking consumers to rank or choose product brands from the choice options of 1 to 4, choosing a 1 for the brand most liked, 2 for the next liked brand, and so on. Even with numerical values, one cannot state how much one brand is preferred to another. In an interval scale, subjects may also be asked to rate or rank a brand preference with choice 1 equal to very high preference, 2 equal to high preference, and so on. The difference between the ordinal and interval scales is that the interval scale will also include a defined, proportional interval between the variables (selections), to determine the degree among choices [6]. Based on these explanations, the UPDRS is most similar to an ordinal scale, as the degree of difference among the choices in unknown.

This observation and conclusion are supported by the literature. The UPDRS choices are ordinal with responses of normal, mild, moderate, and severe, assigned to counting (ranking) numbers that are a label, not a numerical value [7]. The differences between choices, i.e., severity levels, are unknown as there is no known, quantifiable equal distances between them. A score of 4 does not indicate twice the severity as a 2. This must be considered when using these data for statistical analyses, as only average values, along with frequency determinations can be determined from these scores [7].

In addition, when choice meanings are different, one cannot compare the summations or totals as there is no consistency. Choice 1, for example, would always refer to mild in every question. In addition, the same term appears in more than one choice, as in question 1, where the term, severe, is listed in both choices 3 and 4. Do the descriptions provide a difference in their meaning? Would a patient or caregiver understand the difference(s) in these choices? A choice can also list a variety of symptoms and descriptions. In question 1, choice 2, moderate and mild are both listed. Are these options within 2, meaning you may have a moderate or mild symptom level? In addition, there is no information in the scale instructions regarding how the scale choices or score(s) correspond to the stages of symptoms.

It was referenced in [7] that clinical survey questions are difficult to understand, covering many topics in one question; responses are based on question and choice interpretation and the assessment occurs during a brief, clinical visit. There are differences among scale examiners, which affects the score outcomes. Patients may not answer appropriately for fear of the score outcome and to reduce the scores. Other factors may affect these ratings, such as diet, fatigue, or stresses that may alter PD manifestations, but are not reported. [7]. While it was noted that the UPDRS has wide utilization and provides a comprehensive coverage of motor symptoms, there are weaknesses in the scale, which include ambiguities in the text, inadequate instructions for raters, metric flaws, and the absence of screening questions for the nonmotor aspects of PD, as it cannot be used as an adequate, severity measure or diagnosis of any of behavior(s), such as depression or dementia [5].

In addition, there are interrater differences among examiners. The interrater reliability of advanced practice nurse and neurologist neurological assessments of the Unified Parkinson's Disease Rating Scale-Motor Exam (ME) portion was studied in [8]. It was discovered that there was significant agreement between advanced practice nurses and neurologists on the mean motor ratings, but only moderate agreement for whether all motor exam items were normal.

Additional limitations of the UPDRS include the unevenness in the type of information it gathers as bradykinesia-related items are overrepresented in comparison to tremor and postural stability, and the scale contains redundancy in information gathered among the activities of daily living and motor sections, not providing consistency and increasing the administering time [5]. The section constructs are different with a mixture of choices from 0 to 4 and yes/no. The scale is also limited in its utility in the early disease stages where impairments are subtle. To address this issue, some studies have added 0.5 ratings and anchors such as may be normal for healthy elderly subjects, but these modifications have not been validated. The meaning of minimal, mild, and severe stages of PD has not been defined. In addition, insufficient information is available on the ability of the UPDRS to discriminate between disease categories of clinical pertinence. If a measure were used to define these clinical categories, UPDRS scores could be tested to see how consistently they increase as the disease advances clinically [5].

UPDRS Part II is considered culturally biased, as the descriptions for some item ratings are not applicable to all ethnic environments [5]. For example, the dressing item describes difficulty with buttons, even though all cultures do not use them. The cutting and handling utensils item assumes that food is regularly cut for eating and utensils are used, although some cultures serve food in bite-size portions and others do not use utensils. Although the scale was considered applicable to most international urban settings, the UPDRS may be limited by ambiguities when applied to rural and geographically isolated cultures. This points to the need to understand how age, gender, race, and ethnicity affect the UPDRS ratings [5].

The coexistence of diseases, such as diabetes, stroke, and arthritis, can confound the evaluation of PD-related impairment and disability [5]. In addition, coexistent of disorders, such as depression, can potentially affect the speed of a patient's movement, alter motivation, and enhance perceptions of disability even if PD symptoms are stable. How to accommodate these various issues of comorbidities does not exist in the current UPDRS, even though handling for comorbidities is an important asset of a future scale modification [5]. In addition, there is no information in the scale instruction regarding how the score(s) correspond to the stages. These scale gaps explored above are summarized in Table 1.

### 3.2. MDS-UPDRS

The Movement Disorder Society (MDS) developed and published the MDS-UPDRS in 2008. The four parts are retained, but the MDS-UPDRS focuses on the symptoms' impact, not its presence. In addition, the MDS-UPDRS shifts the responses from mild, moderate, and severe in the UPDRS to slight, mild, moderate, and severe. Slight refers to symptoms with low frequency or intensity which causes no impact on function. Mild refers to symptoms which cause a modest impact on function. Moderate refers to symptoms that frequently or intensely impact but do not prevent function whereas severe refers to symptoms that prevent function [4].

**Figure 2:**
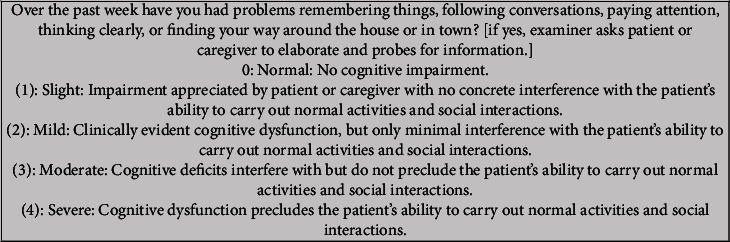
MDS-UPDRS Part I question 1.1 [4].

Part I covers ‘‘nonmotor experiences of daily living” and Part II covers ‘‘motor experiences of daily living'‘. Several questions from Part I and all questions from Part II are designed in a patient/caregiver questionnaire format. The remaining Part I questions regarding complex behaviors and all questions in Part IV regarding motor fluctuations and dyskinesias require an investigator interview. Part III remains as motor examination and Part IV is motor complications [4].  Figure 2 displays question 1.1 from Part I.

Utilizing the example question in Figure 2, one can detect the following:

- Response choices are in a 5-point range (0–4), similar to a Likert scale
- A choice can list a variety of symptoms and descriptions
- Choice numbers do not correspond to a stage

There is an improvement in that the choice meanings are consistent with slight equal to 1, mild equal to 2, and so on. As in the UPDRS, a variety of symptoms and descriptions are provided in the choices. Do the descriptions provide a difference in their meaning? In addition, the MDS-UPDRS is an ordinal scale, as the degree of difference among the choices is unknown. In addition, there is no information in the scale instructions regarding how the scale choices or score(s) correspond to the stages of symptoms.

It was noted in [9] that PD assessment scales are subjective, based on patient self-assessment, and only provide estimates of conceptual, nonobservable factors, with scores on an ordinal scale. Successive survey categories do not represent equal differences of a measured attribute, and the resulting data are ordinal, nonmetric, and categorical, and valid statistics for this type of data are average, mode, median, and frequency distributions [6].

As part of the development of the revised MDS-UPDRS, the MDS Task Force published the new scale along with explanations of its development and testing. Only native English-speaking raters and patients participated in the testing of the new scale. Other than non-Latino Caucasians, not enough participants in any one racial or ethnic group were part of the scale evaluation, preventing statistical analyses within any specific subgroup. Additional steps include non-English scale translations and testing the scale for responsivity to change over time. Interrater reliability needs to be established for the MDS-UPDRS. Factor analysis confirmed that the combined parts do not have a stable factor structure, but when each part is considered separately, the factor structures are clinimetrically sound and clinically pertinent. The recommendation is that the score sections are reported separately and do not collapse into a Total MDS-UPDRS score [4]. The scale gaps explored above are summarized in Table 2.

### 3.3. Practitioner Views on Clinical Scale Use

As part of an Internet search to find published information on the meaning of the PD clinical scale scores, interesting message posts were discovered. A neurosurgeon posted on the ResearchGate website in 2013 the following “What is the best way to track disease progression in Parkinson's disease? I know UPDRS is the standard rating for clinical follow up. I am interested in any knowledge about imaging data or other means to quantify disease progression that is independent of clinical evaluation.” In addition to the original poster asking if there are methods to quantify disease progression, the subjectiveness and inability of the clinical scales to define disease progression were also discovered in the response posts of other clinicians [11]:

- “As for clinical progression, I am not convinced that any available scales cut it in terms of actual disease progress.”
- “The advantages of UPDRS and nonmotor scores are that they are closer linked to patient function and quality-of-life but most PD clinicians know that these are imperfect, insensitive, subjective rating scales…”“.

## 4. Quantitative Results and Discussion

This section provides quantitative analysis of the UPDRS and MDS-UPDRS scores.

### 4.1. Section Scores and Ratios

**Figure 3:**
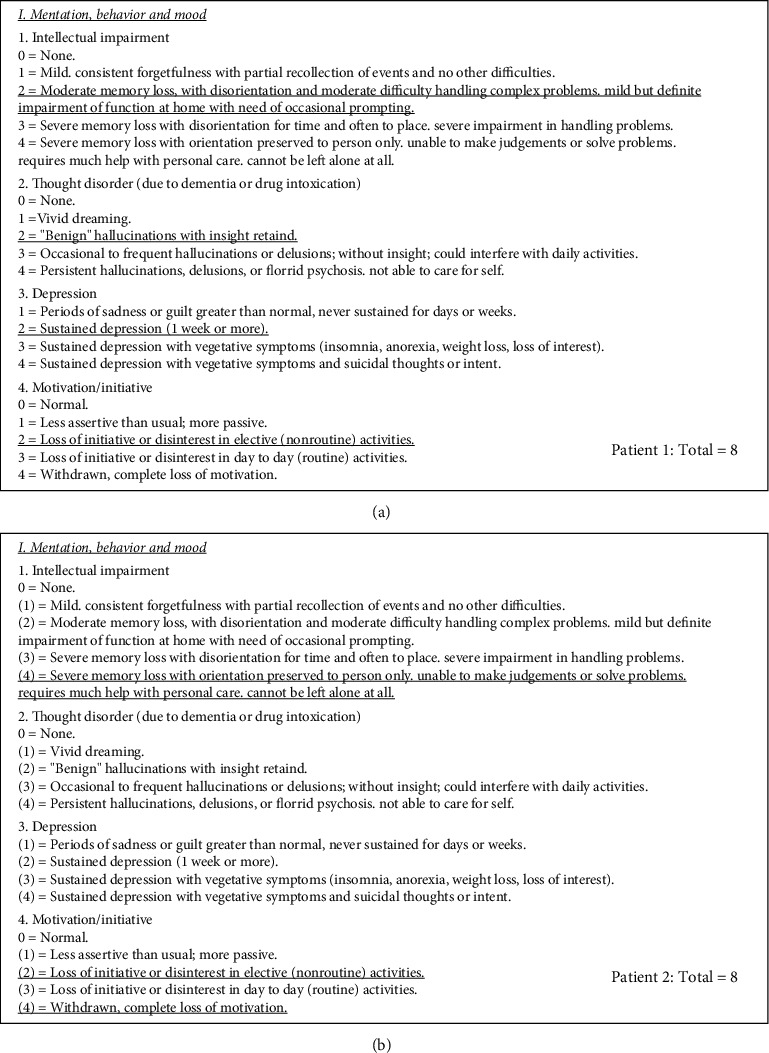
Illustration of same totals with choice combinations in the UPDRS part I (scale from [10]). (a) Patient 1: total = 8. (b) Patient 2: total = 8.

If two patients receive the same score for a section or total, but their choices differ, their disease signatures are not the same. This example is displayed in Figure 3 with UPDRS Part I questions. There is a combination of choices that can provide the same total per section and per entire total, and this needs to be understood. This prevents the use of scores to compare patients.

In cluster analysis studies, PD patients are grouped by similar clinical variable values. In these studies, PD progression was calculated by dividing the UPDRS clinical score by the disease duration. Two studies [12, 13] calculated PD progression as total-UPDRS/disease duration, whereas [14] calculated progression as motor-UPDRS/disease duration. Utilizing Figure 3, with two patients with the same score of 8, even though the selections are different and with the assumption of the same disease duration of 2 years, then, progression would calculate as 8/2 = 4. But, would their progression be considered the same? The numerical totals and ratio results are the same, but the choice selections differed, so the answer would be no. In addition, what does this 4-value mean? Is this labeled as 4 scale scores per year? Is the assumption these patients will gain 4 points per year from the scales? This may not be true if this is the first attempt at filling out the scale.

In addition, the ratio does not convert to a known, quantifiable ratio such as miles per gallon for fuel consumption. How does 4 points per year describe progression and severity? An increase in points may lead one to the conclusion that the disease has progressed or symptoms are more severe, but the degree is unknown with an ordinal scale. As the score summations meaning(s) are unknown, so are their use in ratios.

### 4.2. Score Cutoffs

Furthermore, cutoff values should not be applied to the clinical scales as viewed in Table 3. Severity, cutoff levels of mild, moderate, and severe, for the MDS-UPDRS score sections were proposed in [15]. These mild/moderate and moderate/severe proposed values were (1) Part 1 : 10/11 and 21/22, (2) Part 2 : 12/13 and 29/30, (3) Part 3 : 32/33 and 58/59, and (4) Part 4 : 4/5 and 12/13. But these cutoff values do not apply because a combination of choices can occur for each section total. A patient could select 4 (severe) for two questions and select 0 for the remaining questions, receiving a total score of 8 for Part I. This can be viewed in the table below, for MDS-UPDRS Part I. Eight is less than the 10/11 mild/moderate cutoff, but two, selected severe rankings may not equate to the mild/moderate level. This points to the need in reviewing each selection and not section totals or an overall total, as it is unknown how the summations came about.

### 4.3. Patient Views on Clinical Score Meaning

A PD patient forum was discovered during an Internet search. The Parkinson's Foundation website contains public blogs for people with PD to post questions and comments. The misunderstanding and confusion of the score meanings is highlighted in the following post threads [16]:

- “Does anyone know if there is a “key” to describe what the total UPDRS score indicates? I looked at an online UPDRS calculator at and my result was 42. It says that 0 is no disability and 199 is the worst disability, but does not elaborate further.”
- “I ranked myself and found I was a 41…I'm going to guess anything below 75 is barely disabled if at all.”
- “I went to the site completed everything as best I could and got a 69. Now I'm not sure what this means…. I really do not have a known basis for comparison.”
- “I do not know what it really means either. Obviously, it's somewhat subjective.”
- Using the MDS-DRS, my total score is also 48, but my motor score is 20 (instead of 23 with the original version. I'm a little disappointed that my total score has increased by 6 points in 2 years, but am happy that the motor score has slightly improved…”“.

Unfortunately, this last individual's comment refers to a possible comparison and one-to-one conversion between the UPDRS and MDS-UPDRS scores, which is not the case as the questions and choices are not identical among the two scales. It is concerning when PD patients question what the scale scores mean when attempting to understand their own disease progression.

## 5. Conclusion

A review into the use and meaning of PD clinical scales scores provided a series of discoveries including the following:

- Unclear connection between clinical scores and disease stages
- Scales and scores are ordinal, nonlinear, categorial, not quantifiable values
- Confusion exists regarding the score meaning(s)
- Misuse exists in defining progression with division by scores
- Cutoff values cannot be applied with the combination of results

Despite the critiques of the scales' ordinal format and subjectiveness, a data-driven, quantifiable solution has not been developed. In order to define and understand Parkinson's disease progression, there needs to be a quantifiable result that defines progression. Studying disease symptoms in clusters may assist in the development of symptom divisions, severities, and grouping of patients which in turn may assist with diagnosis, staging definition and future progression tracking and treatment recommendations that may allow a multitude of symptoms to be addressed. Research is required into the development of accurate tracking tools and metrics for both progression and treatment, as current clinical scales are nonlinear and do not provide a meaningful score nor severity level. Utilizing new methods and ways of thinking may change the landscape of understanding, treating, and tracking the disease path of Parkinson's disease and improve the lives of patients and their caregivers.

## Figures and Tables

**Table 1:** A summary of the UPDRS limitations [5–8].

Accuracy unknown	Uneven in type of information gathered
Subjective	Contain redundancies in sections
Numerically nonlinear	Choice selections and section constructs differ
Ordinal scale with score meaning, level, differences, and divisions unknown	UPDRS Part II considered culturally biased
Unable to discriminate between disease categories of clinical relevance	Applicable to most, but not all, international urban settings
Scores cannot differentiate nor compare patients	Effects of age, gender, race, and ethnicity on ratings have not been examined
All score sections not reported	Limited utility in early disease stages
Completed in a limited timeframe (clinic visit)	Can be affected by prior patient activities, but not recorded
Questions difficult to read and understand, responses based on interpretation by rater and patient	No connection to clinical stages
Differences exist among interrater reliabilities	Inadequate instructions for raters
Based on physician's experience, inexperience	Respondents may not answer appropriately, fear of score outcome, lower total scores
Anxiety, sleep disorders, fatigue, urinary disfunction, other symptoms not included	Nonmotor symptoms not clinically diagnosed
How to track comorbidities does not exist	Cannot be used as a severity measure of any behavior

**Table 2:** A summary of the MDS-UPDRS limitations [4, 9, 10].

Accuracy unknown	How to track comorbidities does not exist
Subjective, nonlinear	Low internal consistency computed
Ordinal scale with score meaning, level, differences, and divisions unknown	Need for testing scale for responsivity to change over time
Scores cannot differentiate nor compare patients	Can be affected by prior patient activities, but not recorded
All score sections reported, but what do they mean?	Completed in a limited timeframe (clinic visit)
No connection to clinical stages	Questions difficult to read and understand
Only native English-speaking rated and patients participated in initial testing	Responses based on interpretation by rater and patient
Not enough participants in any other racial or ethnic group in evaluation (other than non-Latino Caucasians)	Respondents may not answer appropriately, fear of score outcome, lower total scores
Effects of age, gender, race, and ethnicity on ratings have not been examined	Based on physician's experience, inexperience
Need for non-English scale translations	Nonmotor symptoms not clinically diagnosed
Interrater reliability needs to be established	Cannot be used as a severity measure of any behavior

**Table 3:** Illustration of cutoff values for MDS-UPDRS Part I (scale from [4]).

Part I		
1.1	Cognitive impairment	—
1.2	Hallucinations and psychosis	—
1.3	Depressed mood	—
1.4	Anxious mood	—
1.5	Apathy	—
1.6	Features of DDS	—
1.6a	Who is filling out questionnaire	LJ patient
		D caregiver
		D patient + caregiver
1.7	Sleep problems	—
1.8	Daytime sleepiness	—
1.9	Pain and other sensations	—
1.10	Urinary problems	4
1.11	Constipation problems	4
1.12	Light headedness on standing	—
1.13	Fatigue	
Total score		8

## Data Availability

No data were used to support this study.
